# Roles of m5C RNA Modification Patterns in Biochemical Recurrence and Tumor Microenvironment Characterization of Prostate Adenocarcinoma

**DOI:** 10.3389/fimmu.2022.869759

**Published:** 2022-05-04

**Authors:** Zhipeng Xu, Shuqiu Chen, Yuxi Zhang, Ruiji Liu, Ming Chen

**Affiliations:** ^1^ Department of Urology, Affiliated Zhongda Hospital of Southeast University, Nanjing, China; ^2^ Surgical Research Center, Institute of Urology, Southeast University Medical School, Nanjing, China; ^3^ Department of Radiation Oncology, The First Affiliated Hospital of Nanjing Medical University, Nanjing, China; ^4^ Department of Urology, Zhongda Hospital Lishui Branch, Nanjing, China

**Keywords:** m5C RNA modification, biochemical recurrence, tumor microenvironment, immunotherapy, prostate adenocarcinoma (PRAD)

## Abstract

**Background:**

Prostate cancer is the second most common cancer with a high risk of biochemical recurrence (BCR) among men. Recently, 5-methylcytosine (m5C) modification has attracted more attention as a new layer of RNA post-transcriptional regulation. Hence, we aimed at investigating the potential roles of m5C modification regulators in the BCR of prostate adenocarcinoma (PRAD).

**Methods:**

CNV data, mutation annotation data, mRNA expression profiles, and clinical data were downloaded from TCGA and GEO databases. Kaplan-Meier curves analysis, log-rank test, univariate and multivariate Cox regression, and time-dependent ROC curves analysis were performed to evaluate the prognostic factors. Principal components analysis (PCA) was applied to validate the distinction between subgroups. Gene set variation analysis (GSVA) was used to investigate the underlying pathways associated with m5C modification patterns. Single sample gene set enrichment analysis (ssGSEA) was utilized to assess the infiltration of distinct immune cells. Tumor Immune Dysfunction and Exclusion (TIDE) prediction was carried out to assess the potential response to immune checkpoint blockade (ICB) therapy. The m5C modification signature was constructed *via* LASSO Cox’s proportional hazards regression method.

**Results:**

After comprehensively analyzing various types of data from TCGA dataset, and exploring the differential expression and prognostic value of each m5C regulator, we identified m5C modification patterns based on 17 m5C regulators. Two patterns presented a significant difference in the risk of BCR, the tumor microenvironment (TME), and immunotherapy response in PRAD. We found that TET2, which was highly expressed in adjacent normal tissues compared to tumor tissues, was closely associated with many infiltrating immune cells. The m5C modification signature was constructed for the clinical application. Risk score calculated by m5C signature was associated with T stage, N stage, Gleason score, and the possibility of BCR (HR, 4.197; 95% CI, 3.016-5.842; p < 0.001). A higher risk score also represented the possibility of immunotherapy response. Finally, the potential roles of m5C modification signature were validated in the testing dataset.

**Conclusions:**

Our study revealed the potential roles of m5C modification in the PRAD BCR and TME diversity, which may provide new insight into the field of prostate cancer in future research.

## Background

Since the 1960s, RNA modifications have been well documented as the key factor in regulating the RNA metabolism under various biological processes (1, 2). Many researchers have recently focused on revealing the specific molecular functions of RNA modifications such as N6-methyladenosine (m6A) (3). With the in-depth research on RNA modifications, 5-methylcytosine (m5C), which was rarely studied, has attracted more attention. M5C has been demonstrated as a widespread modification throughout the coding and non-coding RNA (4, 5). In one report, the m5C modification in the 3’UTR of heparin binding growth factor (HDGF) mRNA can promote the stability of HDGF mRNA, which contributes to bladder cancer progression (6). In another report, Aly/REF export factor (ALYREF) has been identified as a m5C modification binding protein (7). RNA m5C modification is dynamically modulated by relevant proteins in human cells (8). M5C writers are defined as the enzymes contributing to the formation of cytosine-5 methylation, which includes the NOL1/NOP2/SUN domain (NSUNs) family of proteins and the DNA methyltransferase (DNMTs) family members (8, 9). RNA m5C modification demethylases are named as m5C erasers, including the ten-eleven translocator (TETs) family members and Alpha-Ketoglutarate-Dependent Dioxygenase AlkB Homolog 1 (ALKBH1) (10). Moreover, m5C readers, including ALYREF and Y-box binding protein 1 (YBX1), can recognize the mRNA with m5C modification and take part in the biological function of RNA m5C modification (8, 11). In 2017, Xin et al. validated that the m5C reader, ALYREF, regulated mRNA export *via* a m5C-dependent manner (7). Recently, some researchers have also found that ALYREF could stabilize pyruvate kinase muscle isozyme M2 (PKM2) mRNA *via* a m5C‐dependent manner in bladder cancer (12). A previous study found that YBX1 stabilized its target mRNAs *via* an m5C-dependent manner (13). Similarly, it has also been reported that YBX1 could stabilize its target mRNAs by recognizing the m5C methylation site in the HDGF mRNA and by recruiting ELAV like RNA binding protein 1 (ELAVL1) in bladder cancer (6). Previous studies show the need for exploring the relationship between the m5C modification and various biological processes.

It is estimated that prostate cancer is the second most frequent cancer and in 2020 was the fifth leading global cause of cancer death in men (14). Among several histological subtypes of prostate cancer, prostate adenocarcinoma (PRAD) is the most common one (15). With the development of prostate cancer detection and management, patients with prostate cancer at an early stage exhibit a 99% ten-year overall survival (OS) rate (16), whereas more than half of patients undergo biochemical recurrence (BCR) characterized by elevated prostate-specific antigen (PSA) levels after radical therapy (17, 18). It is reported that approximately 35% of patients will experience BCR within 10 years after radical prostatectomy (19, 20). Recently, more and more studies have revealed that the tumor microenvironment (TME), including various immune and non-immune factors, plays an essential role in prostate cancer tumorigenesis and progression (21–24). Exploring the related mechanisms of prostate cancer BCR has become clinically significant. Bora et al. have reported a positive correlation between chronic inflammation and prostate cancer grade (25). Tumor-associated macrophage infiltration can act in prostate cancer as a predictive factor for BCR (26). Some studies have found that regulatory T-cells infiltration is associated with a worse BCR-free survival rate (27, 28). Similarly, it has been demonstrated that higher B-cells infiltration in prostate cancer, and positively correlates with tumor grade and BCR (29). Focusing on the changes in TME may thus conduce to identifying biomarkers for the diagnosis and treatment of prostate cancer.

In this study, we have aimed at clarifying the potential roles of m5C modification regulators inPRAD. By comprehensively analyzing the copy number alteration data, mutation annotation data, and gene expression profiles from The Cancer Genome Atlas (TCGA) database, we could identify the potential pathways related to m5C modification. Based on this, we further explored the association between m5C modification and TME. Subsequently, we constructed an m5C-related signature for assessing the risk of BCR in PRAD and revealed its underlying connection with immunotherapy. This signature was also validated in the external testing dataset (GSE70770). Our results may provide new sights for further research on m5C modification and personalized management of PRAD.

## Methods

### Data Source

In this study, the datasets were obtained from the TCGA and Gene Expression Omnibus (GEO) databases. We downloaded the copy number variation (CNV) data, mutation annotation data, mRNA expression profiles, and clinical data from the TCGA website (https://portal.gdc.cancer.gov/). For the mRNA expression profiles, we converted the FPKM value into the TPM value for further analysis, as it is identical to the microarray values (30, 31). The mRNA expression profiles consisted of 540 samples, including 489 tumor samples and 51 normal samples of which the clinical information was extracted, and BCR was taken as the positive event. After having excluded two samples with uncertain prognostic information, three samples with M1 stage, and four repeated samples, we ultimately included 480 tumor samples and 51 normal samples in the present study. To construct an external testing dataset, we searched the datasets related to PRAD BCR in the GEO database. The GSE70770 dataset, including 200 tumor samples, was eventually selected for its perceived accuracy. The expression matrix and clinical information of GSE70770 dataset were then downloaded and applied to validate the prognostic value of m5C modification signature.

### Analysis of the CNV Data and Mutation Annotation Data

We determined the CNV by using the GISTIC 2.0 software developed by Craig et al. (32). Then, the CNVs of each m5C regulator, including amplification and deletion, were visualized with the R package ‘RCircos’. Mutation annotation format (MAF) of somatic mutation data of TCGA PRAD cohort was applied for further analysis with the R package ‘maftools’ (33).

### Gene Expression Analysis of m5C Regulators and Survival Analysis

According to the relevant literatures, we extracted 17 m5C modification regulators, including 11 writers (NOP2, NSUN2, NSUN3, NSUN4, NSUN5, NSUN6, NSUN7, DNMT1, DNMT3A, DNMT3B, and TRDMT1), four erasers (TET1, TET2, TET3, and ALKBH1) and two readers (ALYREF and YBX1) (8–11). The ‘limma’ package was used to compare the m5C regulators’ expression among different groups. To evaluate the prognostic value, we used the related methods, including Kaplan-Meier curves analysis, log-rank test, univariate and multivariate Cox regression, and time-dependent ROC curves analysis, by the R packages ‘survival’, ‘survminer’, and ‘survivalROC’.

### Identification of m5C Modification Patterns and Molecular Characteristics

The ‘ConsensusClusterPlus’ package was applied to identify the distinct m5C modification patterns in the TCGA-PRAD cohort. According to the relative change in area under the CDF curve and the internal consistency of distinct k values, we determined the number of patterns. In order to assure the stability of the cluster, the step was repeated 1000 times (34). The principal components analysis (PCA) was applied to validate the distinction between different m5C modification patterns with the R package ‘pca3d’ and ‘rgl’ (35, 36).

The gene set variation analysis (GSVA) was performed to investigate the underlying pathways involved in m5C modification using the R package ‘GSEABase’, ‘GSVA’, and ‘pheatmap’ (37). In this study, we downloaded the ‘c2.cp.kegg.v7.2.symbols.gmt’ gene set as the background from the MSigDB database. Differentially enriched pathways were screened out by using the R package ‘limma’ with a threshold of adjusted p-value < 0.05 and │log2(Fold change) │ > 1.

### Evaluation of the Abundance of Various TME Cells

To evaluate the tumor component in each sample, we applied the ESTIMATE algorithm to calculate the score of tumor purity, stromal, immune, and ESTIMATE score in each sample, and the score between different subgroups was further analyzed with R package ‘limma’. The single sample gene set enrichment analysis (ssGSEA) was used to assess the infiltration of distinct immune cells. According to the research of Charoentong et al., the gene set signature, including 18 types of adaptive immune cells and 10 types of innate immune cells, was applied to the ssGSEA analysis (38). This step was conducted and visualized with the R package ‘GSEABase’, ‘GSVA’, ‘ggpubr’, ‘reshape2’, and ‘limma’. The estimated score that was generated in the ssGSEA analysis represented the infiltration of each type of immune cell in each sample.

### Assessing the Differential Expression of Immune Checkpoints, the Response for Immunotherapy, and Drug Sensitivity Analysis

In order to analyze the expression of immune checkpoints in distinct cohorts, we extracted 15 immune checkpoint molecules (GZMA, LAG3, IDO1, CXCL10, CD274, HAVCR2, IFNG, CD8A, TNF, CTLA4, PRF1, PDCD1, GZMB, TBX2, and CXCL9) according to the study of Peng et al. (39). Jiang et al. developed the Tumor Immune Dysfunction and Exclusion (TIDE), a computational method that modeled the tumor immune evasion mechanism, to assess the potential response to immune checkpoint blockade (ICB) therapy (40). TIDE prediction was performed on the website: http://tide.dfci.harvard.edu/. Different expression of immune checkpoint molecules, different microsatellite instability (MSI) score, and different TIDE score were determined with the R package ‘limma’. Moreover, the R package ‘pRRophetic’ was used to analyze the expression profile of TCGA-PRAD cohort in the drug sensitivity analysis (41).

### Construction and Evaluation of the m5C Modification Signature

To construct an m5C modification signature with better prognostic values, we focused on differentially expressed genes (DEGs) between two m5C modification patterns. The DEGs were filters with a threshold of adjusted p-value < 0.001 and │log2(Fold change) │ > 1. Univariate and multivariate COX analysis was used to further select the candidate genes with an independent prognostic value from the DEGs. We then employed the least absolute shrinkage and selection operator (LASSO) Cox’s proportional hazards regression method to screen out the candidate genes with the R package ‘glmnet’ and ‘survival’ (42). The proper genes for the m5C modification signature were selected with the lowest Akaike’s information criterion (AIC) (43). Eventually, the risk score base on the m5C modification signature was constructed with the following formula:


$$\text{Risk score}=\sum_{\text{i}-1}^{\text{n}}\left({\text{Coefficient}}_{\text{i}}\;\times {\text{ Expression}}_{\text{i}}\right)$$

In both the discovery dataset (TCGA-PRAD cohort) and the testing dataset (GSE70770 cohort), the constructed m5C modification signature was evaluated, with the analysis involved in the evaluation as mentioned above. The cohorts were classified into high-risk and low-risk groups with the median risk score as the cut point.

### Clinical Samples and Immunohistochemical (IHC) Staining

The four pairs of PRAD specimens and matched adjacent normal specimens used in our study were collected from The Affiliated Zhongda Hospital of Southeast University, China. Prior informed consent was obtained from all of the patients. This study was approved by the Ethical Committee of the Affiliated Zhongda Hospital of Southeast University for tissue specimens used for research purposes. The 4 μm paraffin-embedded tissue sections were used in IHC staining. The sections were incubated with primary antibodies at 4°C overnight and secondary antibodies conjugated with horseradish peroxidase at room temperature for half an hour. Next, the sections were stained with DAB solution for 10 min and counterstained with hematoxylin for 1 min. Images of the stained sections were obtained with a microscope (Nikon, Tokyo, Japan). The clinical index of four PRAD patients was displayed in **Supplemental Table S1**.

### Statistical Analysis

Rstudio 1.4 and R for windows 4.1.0 software were used to perform the statistical analysis. We applied the Student’s t-test or Wilcoxon test in a two-group comparison of normally or skewed distribution data, respectively. In three or more group comparisons, we used the Kruskal–Wallis test or one-way ANNOVA for parametric or non-parametric comparison purposes. Fisher’s exact test was applied to determine the component differences in subgroups. All statistical tests were two-sided, and p-value < 0.05 was considered statistically significant. ****, p-value < 0.0001; ***, p-value < 0.001; **, p-value < 0.01; *, p-value < 0.05; ns, no significant difference.

## Results

### Genetic Variation of m5C Regulators in PRAD

**Figure 1:**
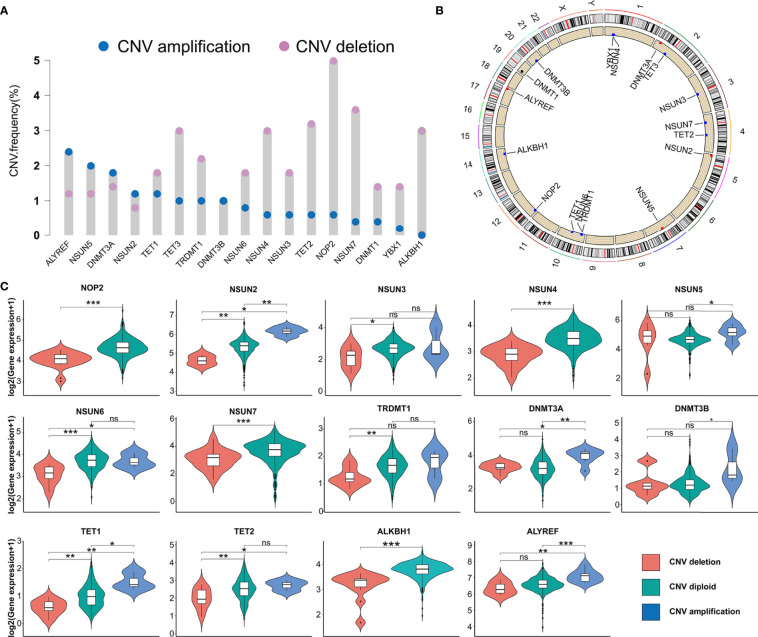
The genetic variation of m5C regulators in PRAD. **(A)** CNV frequency of m5C regulators in TCGA-PRAD cohort. The copy number amplification, blue dot; the copy number deletion, pink dot. **(B)** Location of CNV of 17 m5C regulators on chromosomes in PRAD. **(C)** The association between the CNV and gene expression of m5C regulators in PRAD. ***p-value <0.001; **p-value < 0.01; *p-value < 0.05; ns, no significant difference.

In this study, we evaluated 17 m5C regulators including 11 writers, four erasers, and two readers. The landscape of CNV and somatic mutations and m5C regulators was analyzed in PRAD. CNV was determined as amplification (segment mean > 0.2), diploid (-0.2 < segment mean < 0.2), and deletion (segment mean < -0.2). As shown in **Figure 1A**, ALYREF, NSUN5, DNMT3A, and NSUN2 had a higher frequency of copy number deletion, while a higher frequency of copy number amplification was identified in the other m5C regulators. **Figure 1B** shows the location of CNV of 17 m5C regulators on chromosomes in PRAD. Then, we explored the relationship between CNV incidence and mRNA expression of m5C regulators, which verified that CNV played an essential role in regulating the expression of m5C regulators. As shown in **Figure 1C** and **Supplementary Figure S1A**, CNV amplification was significantly associated with a higher gene expression of ALYREF, DNMT3A, DNMT3B, NSUN2, NSUN5, and TET1. In turn, CNV deletion was significantly associated with a lower gene expression among most m5C regulators. Additionally, the mutations of m5C regulators were identified in only 13 samples among 484 samples in the TCGA-PRAD cohort (**Supplementary Figure S1B**). Considering the frequency of CNV and somatic mutations of m5C regulators was overall low, this indicated that m5C regulators could be conserved in PRAD.

**Figure 2:**
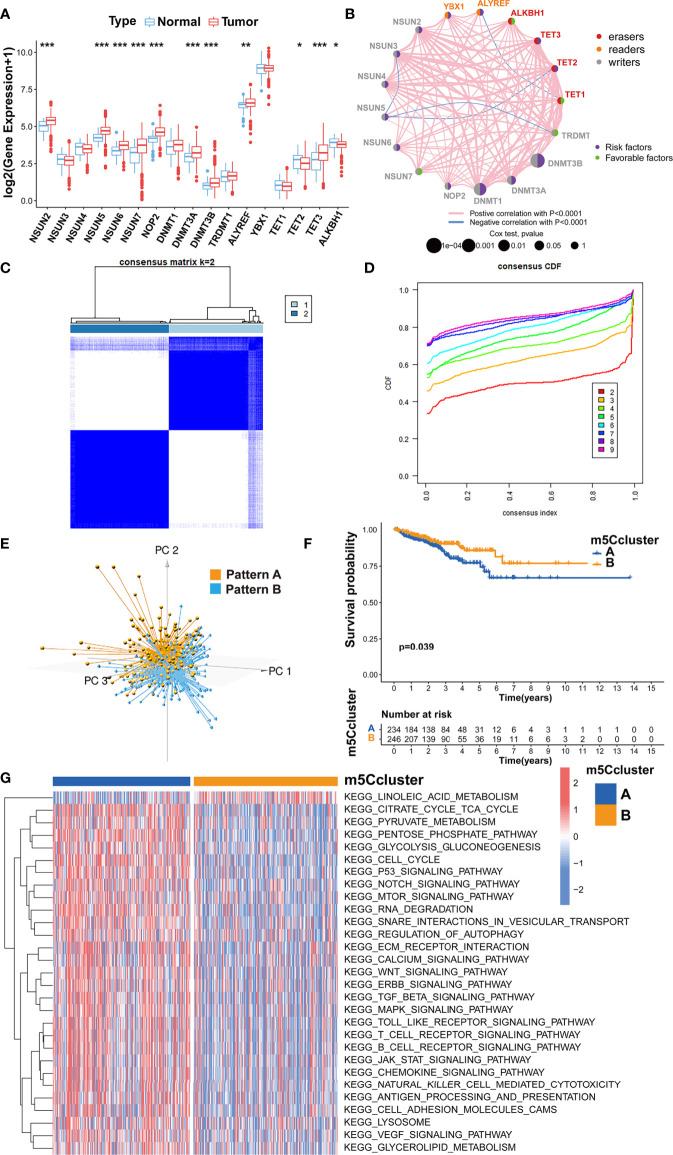
Evaluating the m5C modification patterns based on 17 m5C regulators. **(A)** Gene expression of m5C regulators in PRAD tissue and normal prostate tissue. ***p-value < 0.001; **p-value < 0.01; *p-value < 0.05; ns, no significant difference. **(B)** Gene network of m5C regulators. **(C)** M5C modification patterns identified with K-means clustering based on m5C regulators. **(D)** The CDF curve of the clustering result. **(E)** PCA analysis on the distinction between two m5C modification patterns. **(F)** Kaplan-Meier survival analysis for two m5C modification patterns based on the BCR data of TCGA-PRAD cohort. **(G)** GSVA enrichment analysis determines the distinct biological process and potential pathways between two m5C modification patterns. Red, activated pathways; blue, inhibited pathways.

The analysis of the gene expression in PRAD tissue and normal prostate tissue validated that most m5C writers and readers were highly expressed in PRAD, while the expression of erasers except TET1 and TET3 was significantly lower in PRAD (**Figure 2A**). Besides, the PCA analysis was performed and visualized by the prCOMP and pcd3D algorithm. As shown in **Supplementary Figure S1C**, the 17 m5C regulators could make a clear distinction between the normal and tumor samples in the TCGA-PRAD cohort. The results above indicated that the different expression profiles and genetic variation landscape of m5C regulators could play an important role in PRAD tumorigenesis and progression.

### Evaluation of m5C Modification Patterns Based on 17 Regulators

Kaplan-Meier survival analysis verified that the PRAD patients with a higher expression of NSUN2, NSUN5, NSUN6, NOP2, DNMT1, DNMT3A, DNMT3B, ALYREF, YBX1, TET2, or TET3 could show higher BCR incidence, while higher expression of TRDMT1, TET1 and ALKBH1 could be a good prognostic factor for BCR in PRAD (**Supplementary Figures S2**, **S3**). A univariate Cox regression model was also utilized to evaluate the predictive values of m5C regulators for BCR in TCGA-PRAD cohort (**Supplementary Table S2**). The gene network of m5C regulators depicted their interaction, correlation of mRNA expression, and prognostic values for PRAD patients (**Figure 2B**). These data revealed that most m5C-related genes were the risk factors for BCR in TCGA-PRAD cohort. Unsupervised clustering was performed with ‘ConsensusClusterPlus’ R package to classify patients based on the expression of 17 m5C regulators. Two m5C modification patterns were finally identified, with 234 and 246 patients in patterns A and B, respectively (**Figures 2C, D**). The PCA analysis was utilized to validate the discrimination between two m5C modification patterns (**Figure 2E**). As shown in **Supplementary Figure S4A**, m5C modification pattern A presented higher expression of m5C writers and readers. The survival analysis on BCR indicated that pattern B showed an advantage in non-BCR survival time (**Figure 2F**).

Then, GSVA enrichment analysis was used to reveal the distinct biological process and potential pathways between two m5C modification patterns (**Figure 2G**; **Supplementary Table S3**). Compared to m5C modification pattern B, pattern A was significantly enriched in some pathways associated with the metabolism and function of RNA, such as ‘kegg.RNA.degradation’, ‘kegg.RNA.polymerase’, and ‘kegg.aminoacyl.tRNA.biosynthesis’, which further validated the influence of distinct m5C modification on RNA. We also found that pattern A presented the enrichment of some carcinogenic pathways including p53 signaling pathway, cell cycle, TGF-β signaling pathway, NOTCH signaling pathway, WNT signaling pathway, mTOR signaling pathway, VEGF signaling pathway, MAPK signaling pathway, ERBB signaling pathway, and JAK-STAT signaling pathway. The activation of these pathways could be the reason for the shorter non-BCR survival time in m5C modification pattern A. Besides, it is interesting that pattern A was also markedly associated with immune-related pathways including antigen processing and presentation, chemokine signaling pathway, Toll-like receptor signaling pathway, T cell receptor signaling pathway, B cell receptor signaling pathway, and natural killer cell-mediated cytotoxicity.

### TME Cell Infiltration Characteristics of m5C Modification Patterns

**Figure 3:**
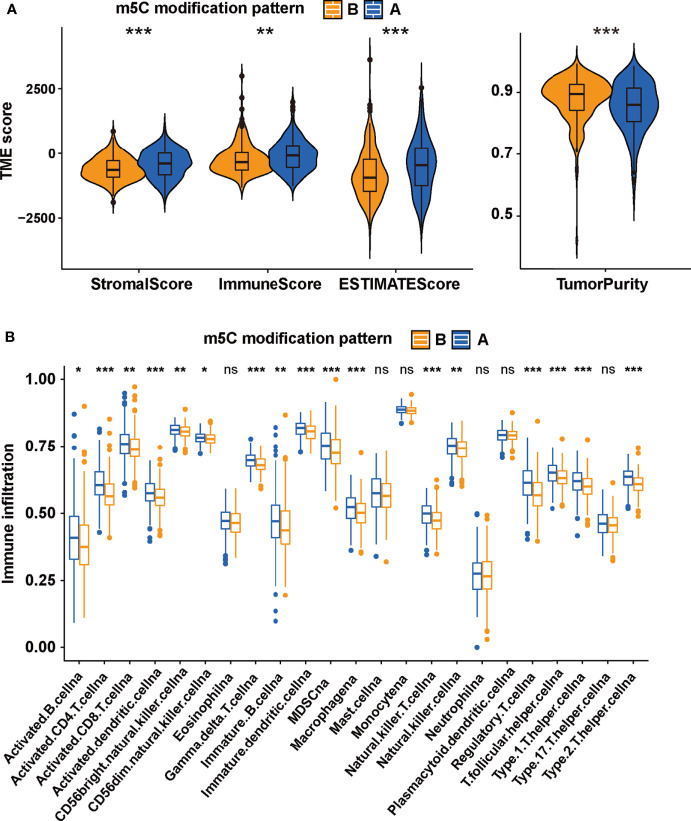
The TME-associated analysis on the m5C modification patterns. **(A)** The tumor purity, immune scores, stromal scores and ESTIMATE scores in two m5C modification patterns based on ESTIMATE algorithm. **(B)** the ssGSEA was performed to evaluate the abundance of diverse immune cells in two m5C modification patterns. ***p-value < 0.001; **p-value < 0.01; *p-value < 0.05; ns, no significant difference.

**Figure 4:**
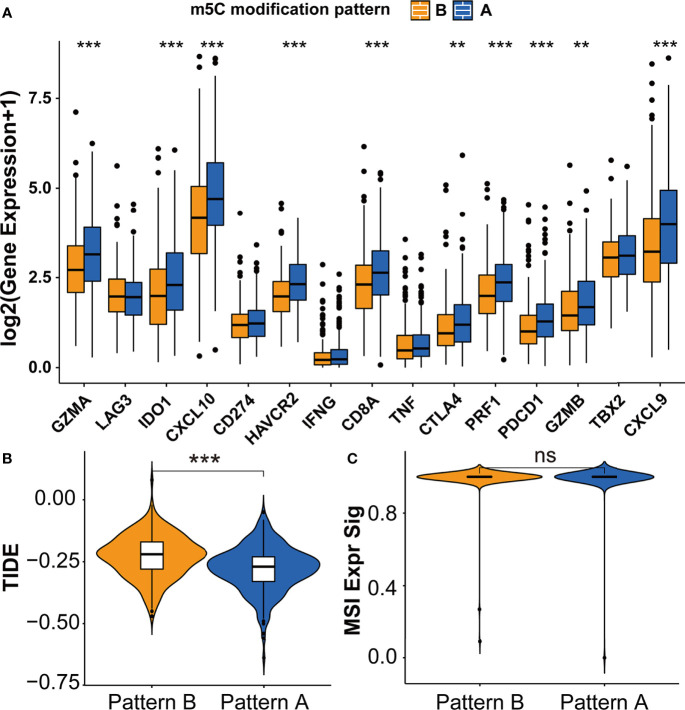
Immunotherapy features in distinct m5C modification patterns. **(A)** The expression of immune checkpoints in two m5C modification patterns. **(B, C)** The predicted immunotherapy response rate of distinct m5C modification patterns based on TIDE analysis. **(B)** TIDE score; **(C)** MSI score. ***p-value < 0.001; **p-value < 0.01; *p-value < 0.05; ns, no significant difference.

The difference between two m5C modification patterns in immune-related pathways prompted the analysis of the TME cell infiltration. The evaluation based on the ESTIMATE algorithm revealed lower tumor purity, higher immune scores, and higher stromal scores of pattern A (**Figure 3A**). We used the ssGSEA algorithm to evaluate the abundance of diverse immune cells in the samples, and the results indicated that pattern A presented a higher abundance of almost all types of immune cells (**Figure 3B**). To explore the potential reason for the contradiction of higher BCR probability and more immune cells infiltration in pattern A, we focused on immune checkpoint pathways which were the inhibitory pathways hardwired into the immune system (44). Now, it has been widely recognized that immune checkpoints were the major mechanism of immune resistance, especially against T cells specific for tumor antigens (45). Hence, we estimated the expression of immune checkpoints in two m5C modification patterns. As shown in **Figure 4A**, immune checkpoints including GZMA, IDO1, CXCL10, HAVCR2, CD8A, CTLA4, PRF1, PDCD1, GZMB, and CXCL9 were higher expressed in pattern A than in pattern B, which indicated that the patients in pattern A could benefit from ICB therapy. Subsequently, we evaluated the immune checkpoints inhibitors (ICI) response by the tumor immune dysfunction and exclusion (TIDE) algorithm. As speculated, immunotherapy was more likely to be effective in patients in pattern A than pattern B according to the TIDE score (**Figure 4B**). There was no significant difference, however, in the MSI score between the two m5C modification patterns (**Figure 4C**). The analysis firmly supported that distinct m5C modification patterns were closely associated with TME and patients’ benefits from ICB therapy. In addition, the pRRophetic algorithm was applied to evaluate the drug sensitivity. Among the drugs identified by R package ‘pRRophetic’, docetaxel was the only drug recommended for clinical treatment of prostate cancer (46). Docetaxel is a kind of tubulin-binding taxanes (47). In 2004, two randomized controlled trials validated that docetaxel was the first chemotherapeutic agent with survival benefits in patients with metastatic castration-resistant prostate cancer (48, 49). As shown in **Supplementary Figure S4B**, the IC50 value of docetaxel was higher in pattern A than in pattern B, which prompted a higher drug sensitivity of docetaxel in pattern B.

### The m5C Eraser TET2 Was Closely Associated With Infiltrating Immune Cells

**Figure 5:**
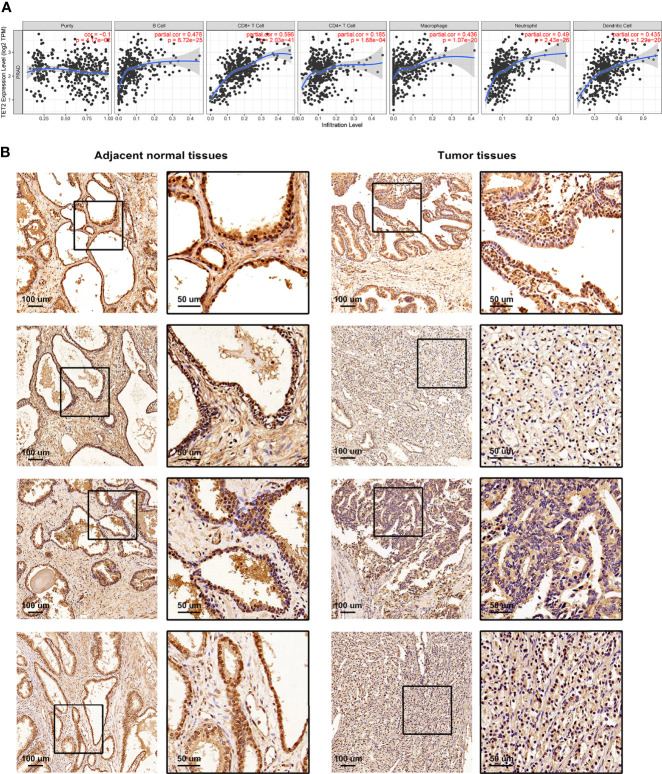
The relationship between TME and m5C eraser TET2. **(A)** The correlation between the expression of TET2 and infiltrating immune cells. **(B)** Representative IHC staining of TET2 in clinical samples.

We performed Spearman’s correlation analysis *via* TIMER algorithm (http://timer.cistrome.org/) to reveal the relationship between TME and each m5C regulator (**Supplementary Figures S5**, **S6**) (50). As shown in **Figure 5A**, the m5C eraser TET2 showed a significant correlation with many immune cells. IHC staining was applied to examine the expression of TET2 in four pairs of PRAD samples and adjacent normal samples. The expression of TET2 was higher in normal prostate epithelial cells than in PRAD cells, which was consistent with our results based on TCGA-PRAD cohort (**Figure 5B**).

### Construction of m5C Modification Signature

**Figure 6:**
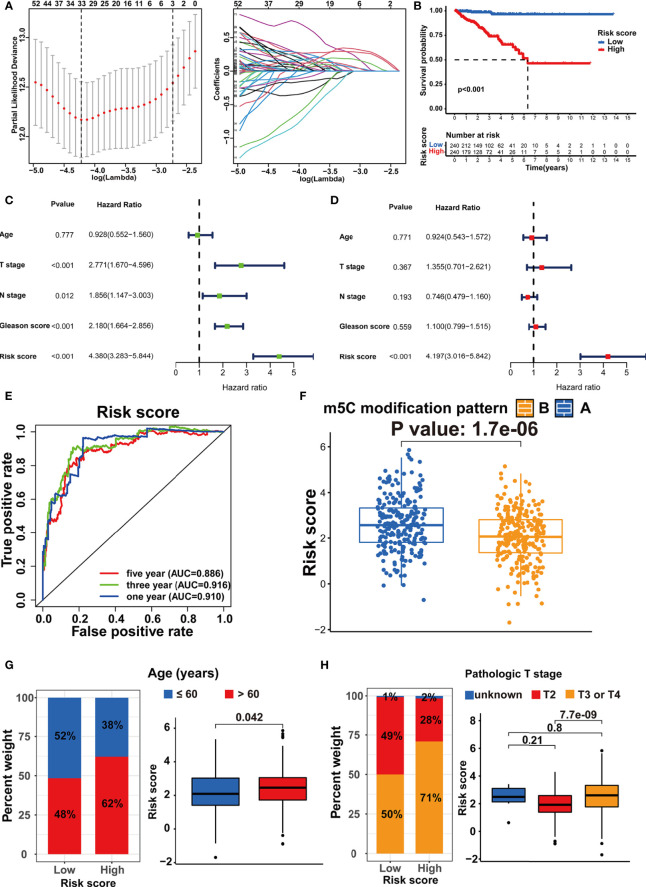
Establishment of the m5C modification signature. **(A)** LASSO coefficient profiles and cross-validation for tuning the parameter selection in the LASSO analysis. **(B)** Kaplan-Meier survival analysis on the association between the BCR and different m5C modification patterns in TCGA-PRAD cohort. **(C)** Univariate COX analysis on the prognostic value of risk score. **(D)** Multivariate COX analysis on the prognostic value of risk score. **(E)** Time-dependent ROC curves analysis for evaluating the prognostic value of risk score in TCGA-PRAD cohort. **(F)** Comparison of the risk score between the m5C modification patterns in TCGA-PRAD cohort. **(G)** The analysis on the association between the risk score and age in TCGA-PRAD cohort. **(H)** The analysis on the association between the risk score and pathologic T stage in TCGA-PRAD cohort.

According to the population-based classification in the TCGA-PRAD cohort, our studies have already validated the prognostic value of m5C modification and the relationship between m5C modification and TME in PRAD. In order to promote the clinical application of m5C modification patterns, we focused on constructing an m5C modification signature for individual tumor cases. We screened out 4877 DEGs (│log2(Fold change) │ > 1 and FDR < 0.001) between two m5C modification patterns (**Supplementary Table S4**). Univariate COX analysis identified 1230 prognosis-related genes out of 4877 genes (**Supplementary Table S5**). Multivariate COX analysis further identified 248 genes with the independent prognostic value as the candidate genes for constructing the m5C modification signature (**Supplemental Table S6**). The LASSO Cox regression algorithm was applied to these candidate genes in the TCGA-PRAD cohort. Eventually, 33 genes were identified based on the minimum criteria to construct the m5C modification signature (**Figure 6A** and **Supplementary Table S7**). The function for calculating the risk score was built according to the coefficients of 33 genes generated by LASSO. As shown in **Supplementary Table S8**, the risk score of each sample in the TCGA-PRAD cohort was calculated, and patients in the TCGA-PRAD cohort were divided into two risk groups according to the median risk score.

Subsequently, the Kaplan-Meier curve analysis and the log-rank test assessed the difference in non-BCR survival time between the two groups (**Figure 6B**). Univariate and multivariate COX analysis indicated the m5C modification signature as the independent prognostic factor for BCR in the TCGA-PRAD cohort (**Figures 6C, D**). The results of the time-dependent ROC curves analysis showed that the AUC for m5C modification signature was 0.910, 0.916, and 0.886 for one-, three-, and five-year non-BCR survival time in the TCGA-PRAD cohort (**Figure 6E**). These results verified that the m5C modification signature was a robust and independent prognostic factor, and this signature presented higher predictive accuracy for BCR than age, pathologic T stage, pathologic N stage, and Gleason score in PRAD. We further validated the association between m5C modification patterns and the signature. As shown in **Figure 6F**, pattern A exhibited a significantly higher risk score than pattern B. Considering that pattern A was associated with shorter non-BCR survival time, the results again verified the prognostic value of the m5C modification signature.

**Figure 7:**
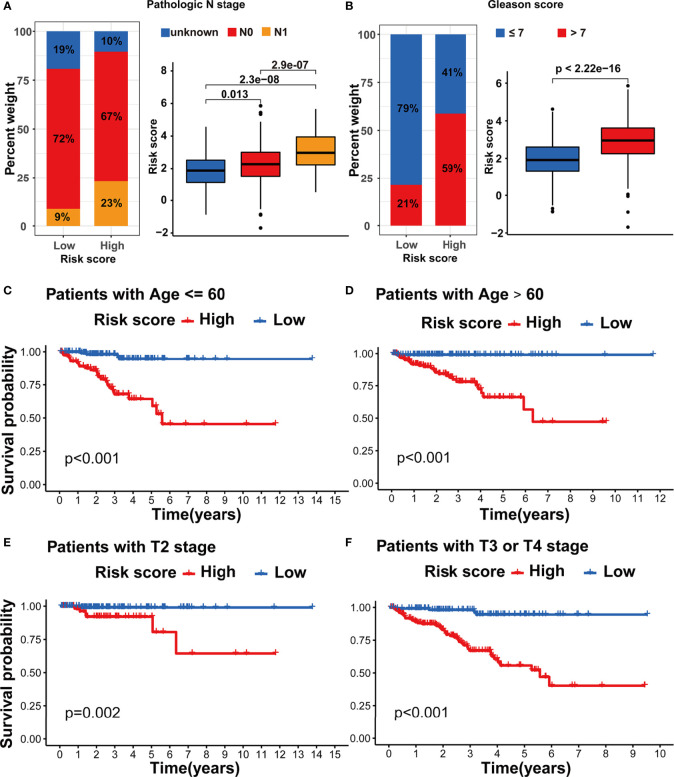
Evaluation of the m5C modification signature in TCGA-PRAD cohort. **(A)** The analysis on the association between the risk score and pathologic N stage in TCGA-PRAD cohort. **(B)** The analysis on the association between the risk score and Gleason score in TCGA-PRAD cohort. **(C-F)** Kaplan-Meier curve analysis in the subgroups with different clinical characteristics. **(C)** patients with age <=60; **(D)** patients with age >60; **(E)** patients with T2 stage; **(F)** patients with T3 or T4 stage.

**Figure 8:**
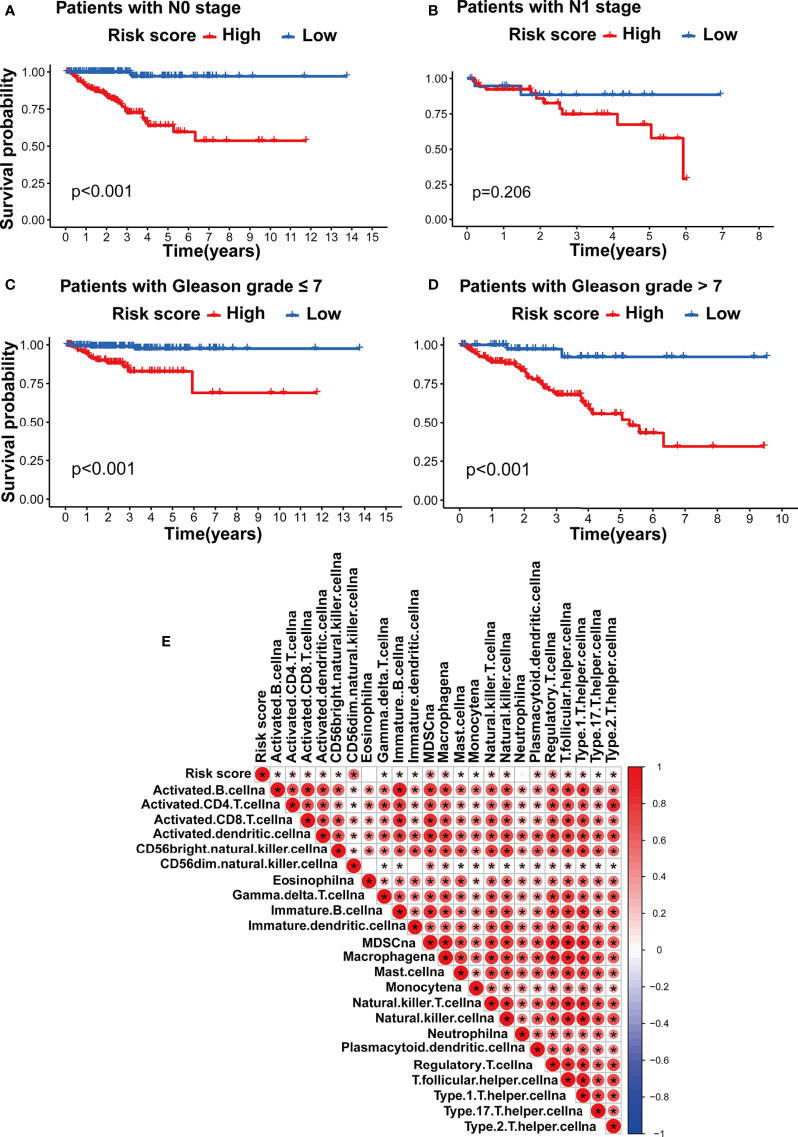
Evaluation of the m5C modification signature in TCGA-PRAD cohort. **(A–D)** Kaplan-Meier curve analysis in the subgroups with different clinical characteristics. **(A)** patients with N0 stage; **(B)** patients with N1 stage; **(C)** patients with Gleason score <=7; **(D)** patients with Gleason score >7. **(E)** Correlations between risk score and immune cell infiltration in TCGA-PRAD cohort *via* Spearman analysis. A negative correlation was marked with blue and positive correlation with red. *p-value < 0.05; ns, no significant difference.

We assessed the association between the m5C modification signature and the clinical characteristics in the TCGA-PRAD cohorts. The results demonstrated that elderly patients, higher Gleason score, advanced T stage, and advanced N stage were significantly associated with a higher risk score (**Figures 6G, H**, **7A, B**). We performed the Kaplan-Meier curve analysis in the subgroups with different clinical characteristics to further evaluate the prognostic value of the m5C modification signature in the distinct population. As shown in **Figures 7C–F**, **8A–D**, the m5C modification signature exhibited a prognostic power in various subgroups except for the patients with N1 stage. In the TCGA-PRAD cohort, there were only 76 patients with N1 stage, which could be the reason for the failure of the m5C modification signature in the N1 stage subgroup. These results demonstrated that the m5C modification signature had the potential to act as a biomarker for assessing the clinical characteristics and predicting the BCR in PRAD patients.

### Correlation of m5C Modification Signature With TME and Immunotherapy

**Figure 9:**
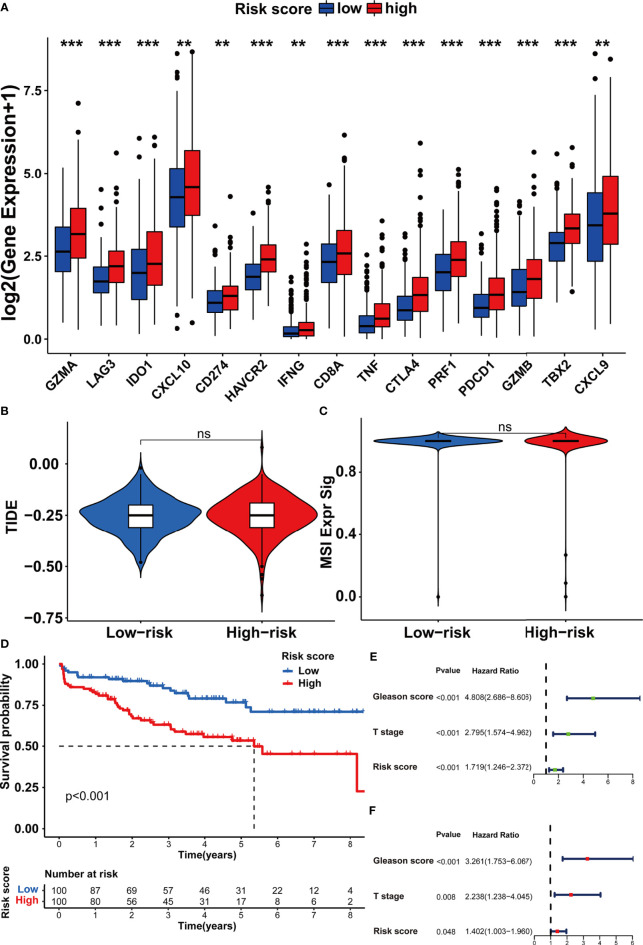
Assessing the immunotherapy features in different risk score groups based on the m5C modification signature. **(A)** The expression of immune checkpoints in high and low-risk groups. **(B, C)** The predicted immunotherapy response rate of high and low-risk groups is based on TIDE analysis. **(B)** TIDE score; **(C)** MSI score. **(D–F)** Survival analysis was performed to evaluate the prognostic value of risk score in the testing cohort. **(D)** Kaplan-Meier survival analysis; **(E)** Univariate COX analysis; **(F)** Multivariate COX analysis. ***p-value < 0.001; **p-value < 0.01; *p-value < 0.05; ns, no significant difference.

In order to investigate the TME characteristics of the m5C modification signature, we determined the association between the m5C modification signature and the infiltration of diverse immune cells (**Figure 8E**). The risk score based on the m5C modification signature was positively associated with the abundance of most immune cells. Subsequently, we analyzed the expression profiles of immune checkpoints in the high- and low-risk score groups. As shown in **Figure 9A**, the expression of all immune checkpoints was markedly higher in the high-risk group. However, we found that there was no significant difference in the TIDE score and MSI score between the high- and low-risk score groups (**Figures 9B, C**).

### Validation of m5C Modification Signature in the Testing Dataset

**Figure 10:**
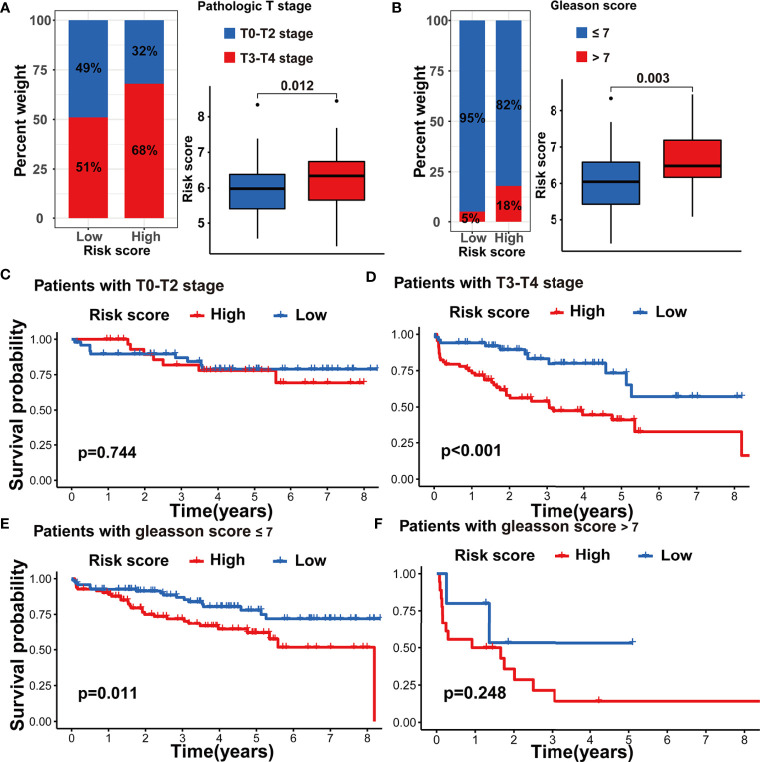
Validation of m5C modification signature in the testing dataset. **(A)** The analysis on the association between the risk score and pathologic T stage. **(B)** The analysis on the association between the risk score and Gleason score. **(C–F)** Kaplan-Meier curve analysis in the subgroups with different clinical characteristics in the testing dataset. **(C)** patients with T0-T2 stage; **(D)** patients with T3-T4 stage; **(E)** patients with Gleason score <=7; **(F)** patients with Gleason score >7.

To further assess the stability of the m5C modification signature, we applied the constructed signature to the other independent PRAD cohorts. The relevant datasets were searched in the GEO database. Eventually, the GSE70770 dataset was chosen as the testing dataset due to its accurate clinical information. According to the function for calculating the risk score, we acquired the risk score for each patient in the testing dataset (**Supplementary Table S9**). By taking the median risk score as the cutoff point, the patients in the testing dataset were divided into two groups. As shown in **Figure 9D**, the patients with higher risk scores were significantly associated with shorter non-BCR survival time. The results of univariate and multivariate COX analysis indicated that the m5C modification signature also had the independent prognostic value for BCR in the testing dataset (**Figures 9E, F**). Furthermore, we performed the analysis of the correlation between the m5C modification signature and clinical characteristics, and **Figures 10A, B** indicated that higher risk score based on the m5C modification signature was closely associated with higher Gleason score and advanced T stage. Moreover, we further evaluated the predictive power of the m5C modification signature in patients with specific clinical characteristics. As shown in **Figures 10C–F** a higher risk score was significantly associated with shorter non-BCR survival time in patients with Gleason score ≤ 7, and the consistent result was validated in patients with T3 or T4 stage.

**Figure 11:**
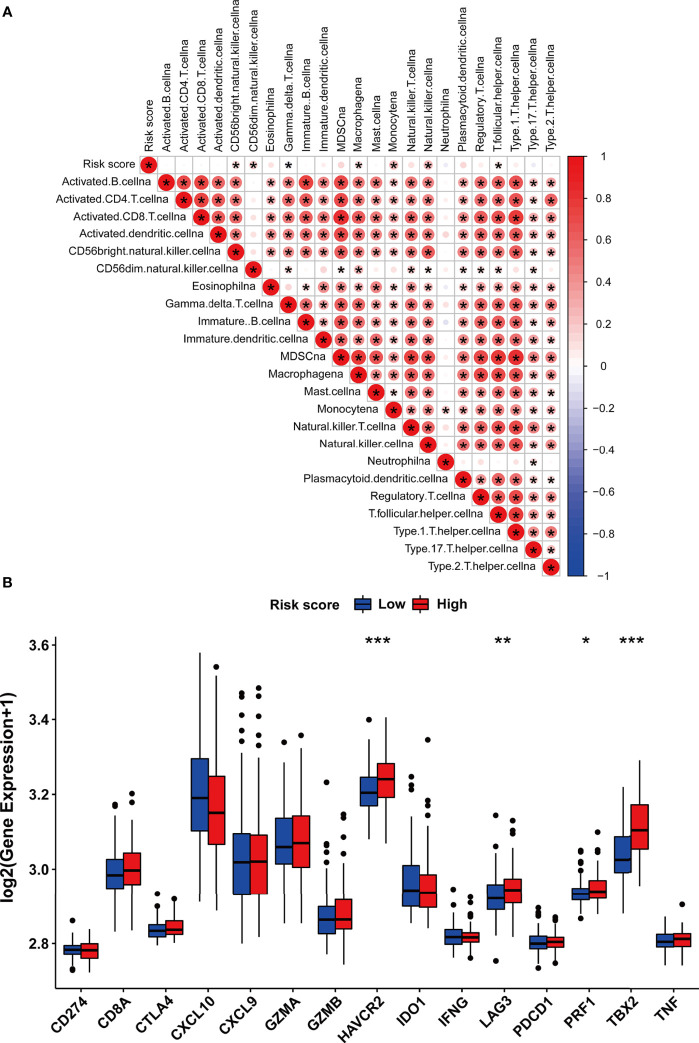
The immune features of m5C modification signature in the testing dataset. **(A)** Correlations between risk score and immune cell infiltration in the testing dataset *via* Spearman analysis. A negative correlation was marked with blue and a positive correlation with red. **(B)** The expression of immune checkpoints in high and low-risk groups. ***p-value < 0.001; **p-value < 0.01; *p-value < 0.05; ns, no significant difference.

Considering the correlation between m5C modification and tumor immune in the TCGA-PRAD cohort, we also analyzed this correlation in the testing dataset. The ssGSEA algorithm was applied to assess immune cells infiltration, and the results validated that the risk score was significantly associated with the abundance of several types of immune cells including natural killer cells, macrophages, monocytes, and so on (**Figure 11A**). Among immune checkpoints, HAVCR2, LAG3, PRF1, and TBX2 presented significantly higher expression in the high-risk group than in the low-risk group (**Figure 11B**). These data indicated that the correlation between m5C modification and tumor immune was not less significant in the testing dataset than in the TCGA-cohort.

## Discussion

Owing to the advance in detection technologies, accumulating research has verified that m5C modification played a key role in the post-transcriptional modification of gene expression, which was closely related to tumor formation, maintenance, and progression (10, 51, 52). As reported, the m5C writer NSUN2 was proven to stabilize the GRB2 mRNA *via* m5C dependent manner in esophageal cancer (53). Some studies have validated that m5C modification played an important role in bladder cancer progression *via* modulating the stabilization of mRNA (6, 12). Moreover, increasing evidence has recently proven that RNA modification regulators had the potential to act as the biomarkers for diagnosis and prognostic monitoring in cancer (54–56). The m5C reader ALYREF expression was dysregulated in several cancers, and higher ALYREF expression was associated with poor prognosis in hepatocellular carcinoma (57). Some studies indicated that m5C writers NSUN3 and NSUN4 were associated with the infiltration of some immune cells, such as CD8+ T cells and neutrophils (58). However, there remained a lack of comprehensive analysis of the prognostic value and functional annotation of m5C regulators in prostate cancer.

In this study, the differential expression of 17 m5C regulators between the normal tissue and tumor tissue was determined. The m5C writers and readers dominantly presented higher expression, while most m5C erasers presented lower expression. Survival analysis verified that m5C writers and readers were predominantly associated with the early BCR in PRAD. These data prompted that the activation of m5C modification may promote the tumorigenesis and progression of PRAD. Two m5C modification patterns based on the 17 m5C regulators were further identified. Interestingly, the two patterns exhibited distinct TME characteristics. Pattern A with higher BCR risk was associated with a higher abundance of immune cells, higher ESTIMATE score, and lower tumor purity. Considering the contradiction between poor prognosis and higher immune cells infiltration, we assumed that the immune checkpoints may contribute to blocking the anti-tumor immune response in pattern A. As assumed, the immune checkpoints showed higher expression in pattern A. Lower TIDE score also suggested the potential response to ICB therapy in pattern A. Besides, pattern A presented higher expression of m5C writers and readers. Hence, although pattern A with higher m5C modification had a worse prognosis, the anti-tumor immunity in the cases in pattern A or with higher m5C modification may be restored after ICB therapy. The data above suggest a potential correlation between m5C modification and immunotherapy, which was clinically significant in the era of immunotherapy and remained to be further validated through experimental research.

Herein, we tried to promote the clinical application of m5C modification. The m5C modification signature was established as a risk score model. Survival analysis verified the robust and independent prognostic value of the m5C signature in both the training dataset (TCGA-PRAD cohort) and testing dataset (GSE70770 cohort). Our study also revealed that the risk score based on m5C signature was closely associated with main clinical characteristics, such as pathologic T stage, pathologic N stage, and Gleason score. Besides, our study verified that a higher risk score calculated by the m5C signature also suggested a higher abundance of TME cells and higher immune checkpoints expression. However, no significant correlation was determined between TIDE score and risk score. Despite this, these results still hinted at the clinical value of the m5C signature in evaluating individual prognosis and TME cells characteristics of patients with PRAD.

However, some limitations are also worth noting. Firstly, the m5C signature did not exhibit a valid predictive value in TIDE score, while a significant difference was verified between two m5C modification patterns. The unexpected results may be attributed to the LASSO Cox regression which mainly preserved prognostic features for constructing the m5C signature. And the immune-related DEGs may eventually not be included in the construction of m5C signature *via* LASSO Cox’s regression method. This disadvantage limited the application of m5C signature in predicting the individual response to immunotherapy. Secondly, the clinicopathological variables, especially immunotherapy-related data, obtained from the public database were not as comprehensive as those involved in clinical practice. Other potential values of m5C modification remain to be further studied, which can further reduce bias in the performance of m5C score signature. Thirdly, the findings in this study were based on bioinformatics research and have not been validated in relevant experiments. In the future, we will conduct *in vitro* and *in vivo* experimental verification of these findings.

## Conclusions

Herein, we revealed the roles of m5C modification patterns in the PRAD BCR and TME diversity. The m5C signature based on distinct modification patterns had the robust and independent prognostic power for PRAD BCR, and could be used to predict the individual response to immune therapy. Our comprehensive analysis of m5C modification can provide new insight into the field of PRAD research, and contribute to the understanding of TME and immunotherapy in the future.

## Data Availability Statement

The original contributions presented in the study are included in the article/**Supplementary Material**. Further inquiries can be directed to the corresponding authors.

## Ethics Statement

The studies involving human participants were reviewed and approved by The Ethical committee of the Affiliated Zhongda Hospital of Southeast University. The patients/participants provided their written informed consent to participate in this study.

## Author Contributions

Conceptualization, ZX and RL; methodology, ZX; software, YZ; validation, ZX, RL, and YZ; formal analysis, ZX and SC; resources, MC; data curation, ZX, RL, and SC; writing—original draft preparation, ZX; writing—review and editing, SC; visualization, ZX, RL, and MC; supervision, MC; funding acquisition, MC. All authors have read and agreed to the published version of the manuscript.

## Funding

This work was supported by the National Natural Science Foundation of China (81872089, 81871157, 82070773, 82102831), The National Key Research and Development Program of China (SQ2017YFSF090096), Jiangsu Provincial Key Research and Development Program (BE2019751), Innovative Team of Jiangsu Provincial (2017XKJQW07) and Natural Science Foundation of Jiangsu Province (BK20201271).

## Conflict of Interest

The authors declare that the research was conducted in the absence of any commercial or financial relationships that could be construed as a potential conflict of interest.

## Publisher’s Note

All claims expressed in this article are solely those of the authors and do not necessarily represent those of their affiliated organizations, or those of the publisher, the editors and the reviewers. Any product that may be evaluated in this article, or claim that may be made by its manufacturer, is not guaranteed or endorsed by the publisher.

## Supplementary Material

Supplementary Figure 1 The genetic variation of m5C regulators in PRAD. **(A)** The association between the CNV and gene expression of m5C regulators in PRAD. ****, p-value<0.0001; ***, p-value<0.001; **, p-value<0.01; *, p-value<0.05; ns, no significant difference. **(B)** The mutations of m5C regulators in 484 patients of TCGA-PRAD cohort. The upper barplot, the TMB of each patient; the right barplot, the number of patients with mutations in specific genes; the stacked barplot, the proportion of conversions in each patient. **(C)** PCA analysis on the distinction between the normal and tumor samples based on m5C regulators.

Supplementary Figure 2 Kaplan-Meier survival analysis on the association between the BCR and gene expression of m5C regulators in TCGA-PRAD cohort (Part I).

Supplementary Figure 3 Kaplan-Meier survival analysis on the association between the BCR and gene expression of m5C regulators in TCGA-PRAD cohort (Part II).

Supplementary Figure 4 The expression of m5C regulators and drug sensitivity analysis in two m5C modification patterns. **(A)** The expression of m5C regulators in two m5C modification patterns. **(B)** Docetaxel sensitivity analysis in two m5C modification patterns.

Supplementary Figure 5 Spearman’s correlation analysis on the relationship between TME and each m5C regulator (Part I).

Supplementary Figure 6 Spearman’s correlation analysis on the relationship between TME and each m5C regulator (Part II).

## Abbreviations

BCR, biochemical recurrence; m5C, 5-methylcytosine; CNV, copy number variation; TCGA, The Cancer Genome Atlas; GEO, Gene Expression Omnibus; PCA, principal components analysis; GSVA, gene set variation analysis; ssGSEA, single sample gene set enrichment analysis;TIDE, Tumor Immune Dysfunction and Exclusion; ICB, immune checkpoint blockade; ICI, immune checkpoints inhibitor; LASSO, least absolute shrinkage and selection operator; TME, tumor microenvironment; m6A, N6-methyladenosine; DNMT, DNA methyltransferase; TET, ten-eleven translocator family; NSUN, NOL1/NOP2/SUN domain family; ALKBH1, Alpha-Ketoglutarate-Dependent Dioxygenase AlkB Homolog 1; PRAD, prostate adenocarcinoma; OS, overall survival; PSA, prostate-specific antigen; MAF,Mutation annotation format; MSI, microsatellite instability; DEGs, differentially expressed genes; AIC, Akaike’s information criterion.
