# Supplementary figures and images for: Roles of m5C RNA Modification Patterns in Biochemical Recurrence and Tumor Microenvironment Characterization of Prostate Adenocarcinoma
Source: Front Immunol. 2022 May 4;13:869759. doi: 10.3389/fimmu.2022.869759 (PMC9114358; doi:10.3389/fimmu.2022.869759)

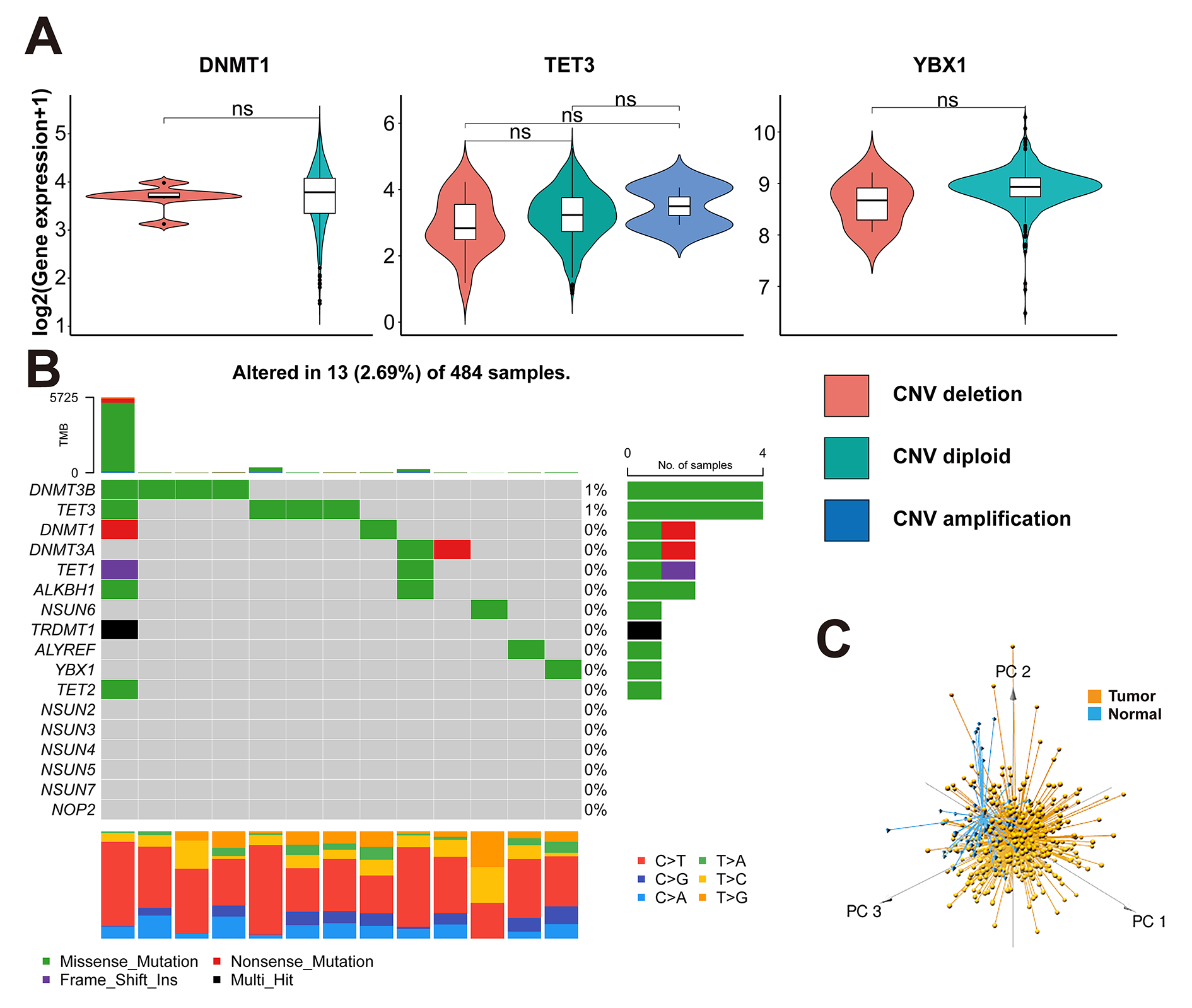

Supplement: Supplementary Figure 1 — The genetic variation of m5C regulators in PRAD. (A) The association between the CNV and gene expression of m5C regulators in PRAD. ****, p-value<0.0001; ***, p-value<0.001; **, p-value<0.01; *, p-value<0.05; ns, no significant difference. (B) The mutations of m5C regulators in 484 patients of TCGA-PRAD cohort. The upper barplot, the TMB of each patient; the right barplot, the number of patients with mutations in specific genes; the stacked barplot, the proportion of conversions in each patient. (C) PCA analysis on the distinction between the normal and tumor samples based on m5C regulators. [file Image_1.tif]

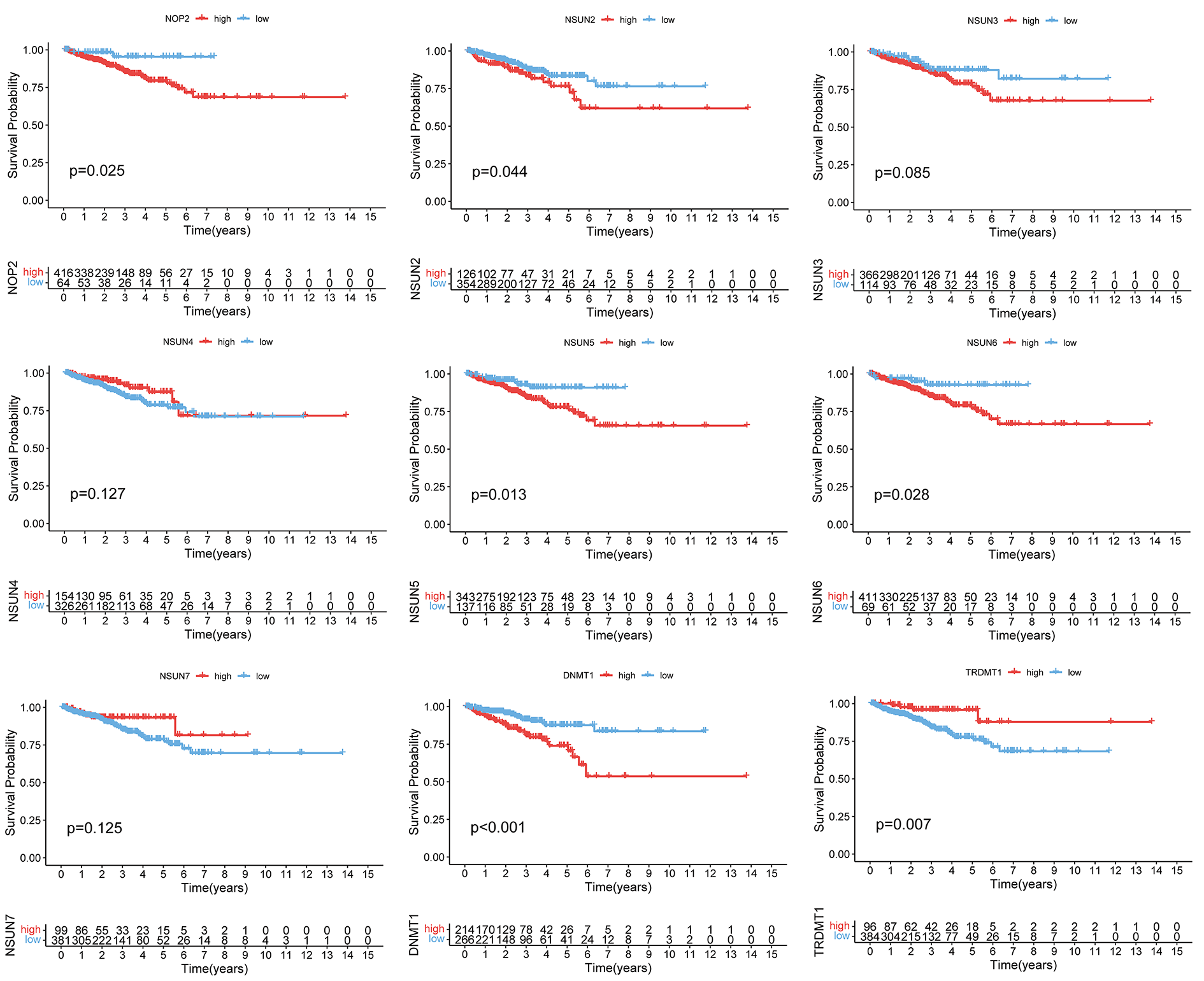

Supplement: Supplementary Figure 2 — Kaplan-Meier survival analysis on the association between the BCR and gene expression of m5C regulators in TCGA-PRAD cohort (Part I). [file Image_2.tif]

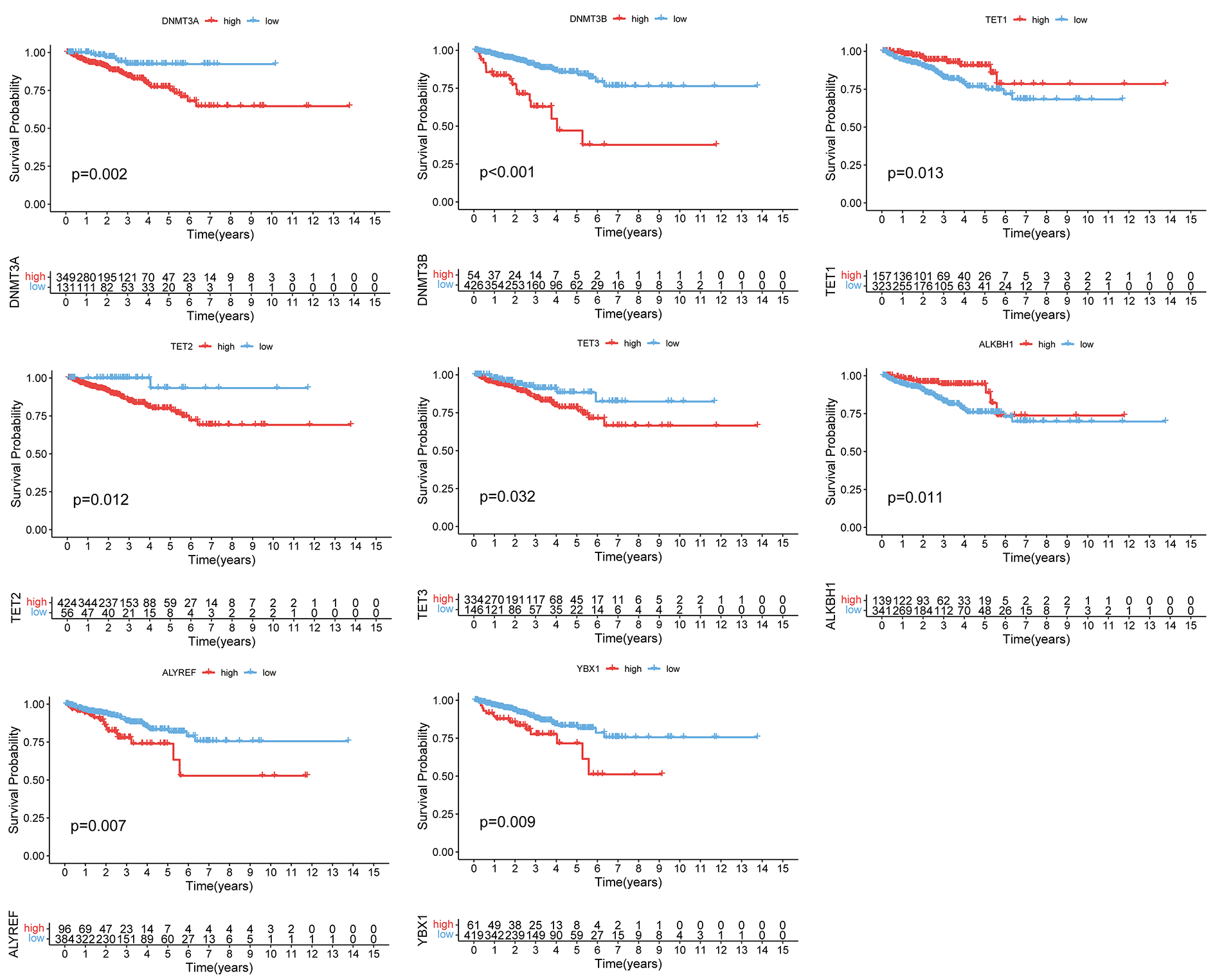

Supplement: Supplementary Figure 3 — Kaplan-Meier survival analysis on the association between the BCR and gene expression of m5C regulators in TCGA-PRAD cohort (Part II). [file Image_3.tif]

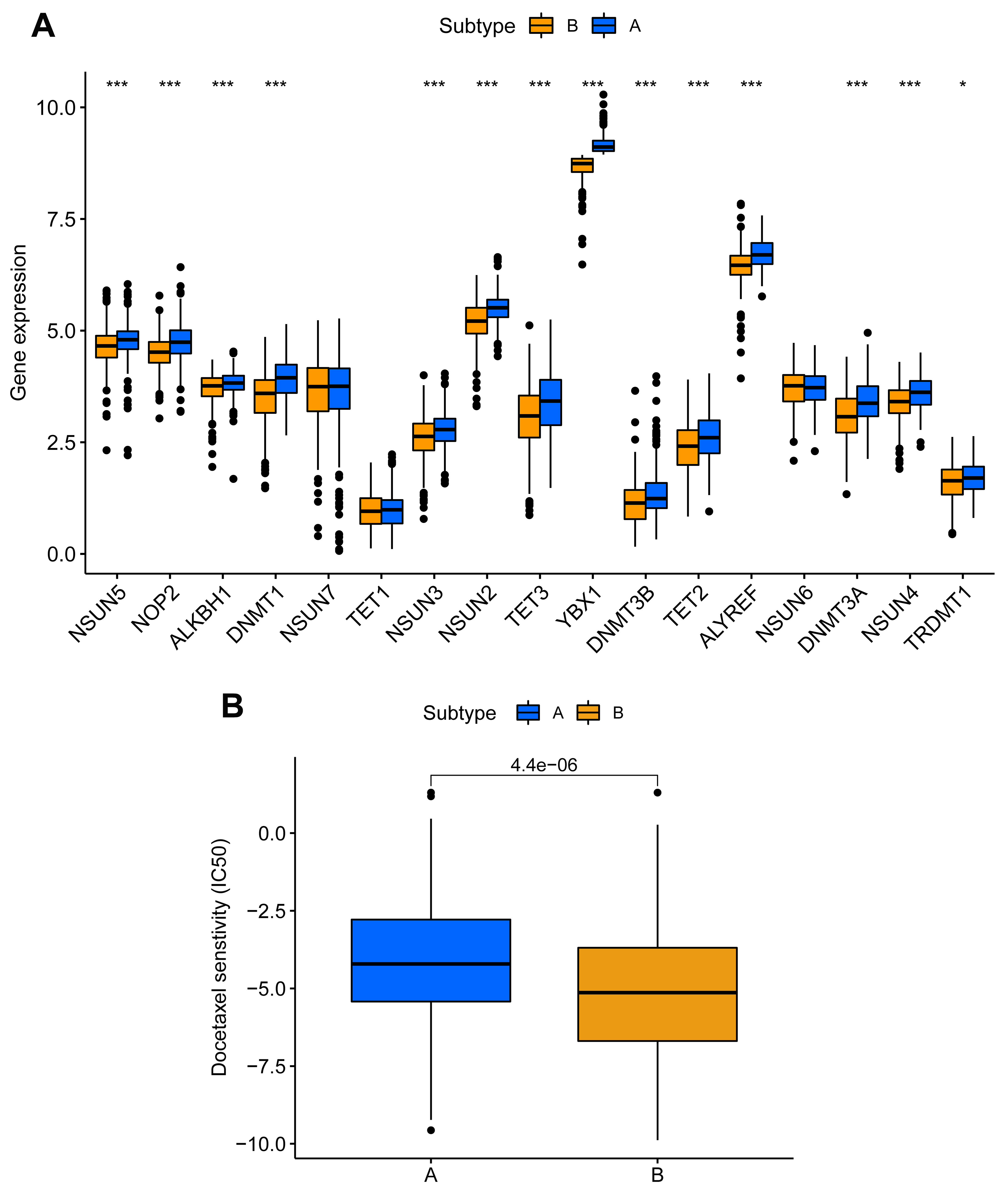

Supplement: Supplementary Figure 4 — The expression of m5C regulators and drug sensitivity analysis in two m5C modification patterns. (A) The expression of m5C regulators in two m5C modification patterns. (B) Docetaxel sensitivity analysis in two m5C modification patterns. [file Image_4.tif]

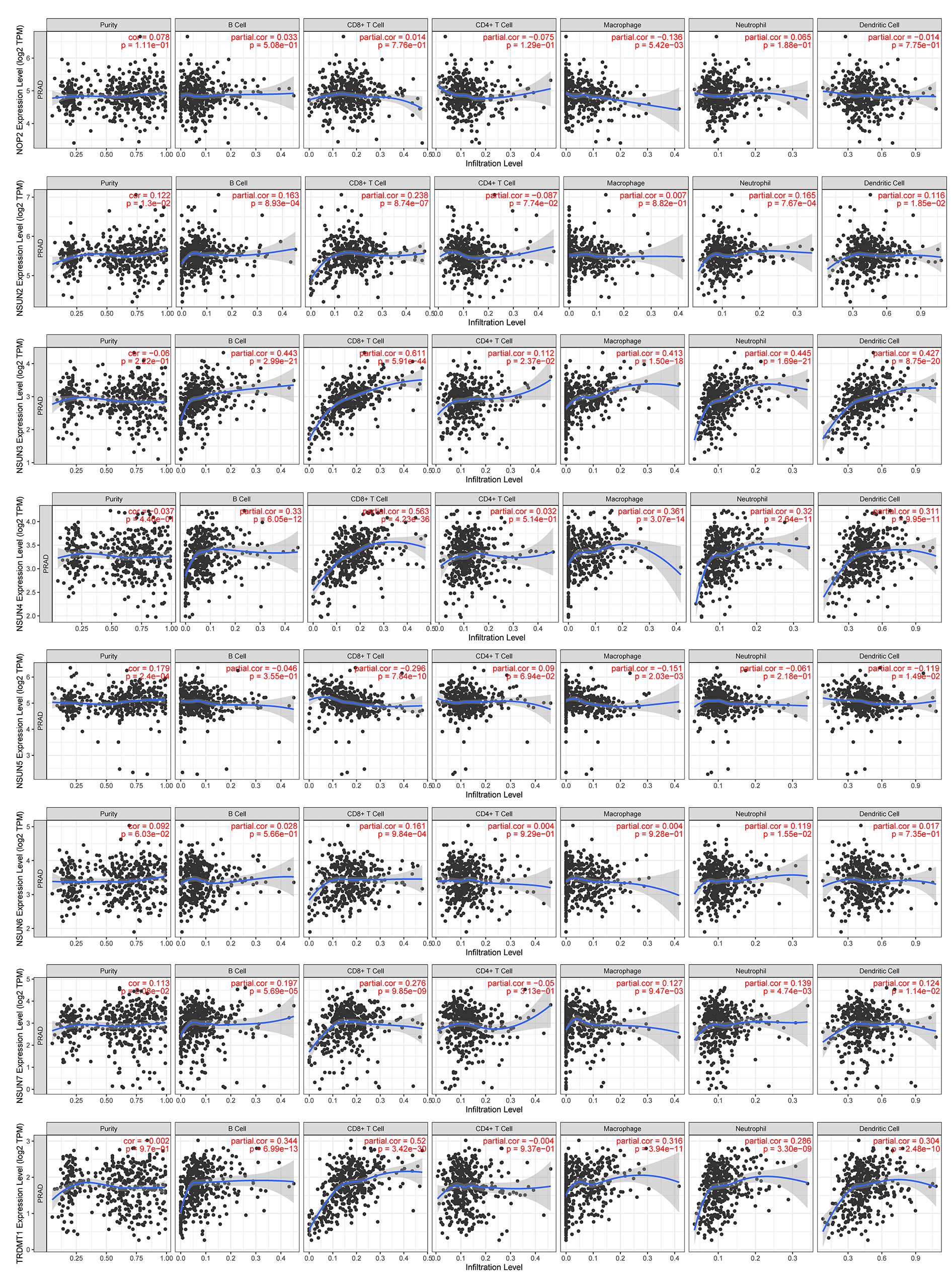

Supplement: Supplementary Figure 5 — Spearman’s correlation analysis on the relationship between TME and each m5C regulator (Part I). [file Image_5.tif]

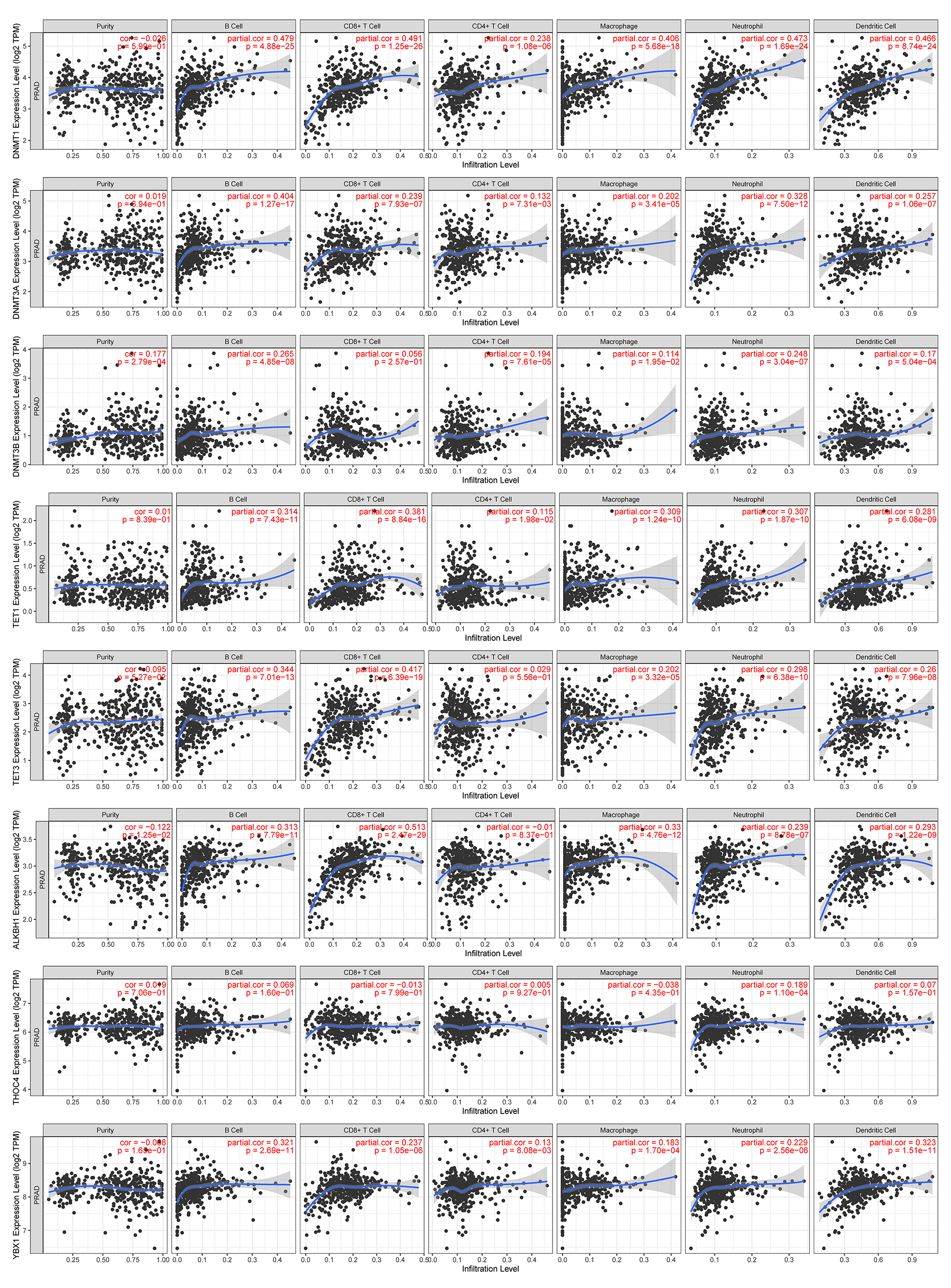

Supplement: Supplementary Figure 6 — Spearman’s correlation analysis on the relationship between TME and each m5C regulator (Part II). [file Image_6.tif]
