# Supplementary material for: Roles of m5C RNA Modification Patterns in Biochemical Recurrence and Tumor Microenvironment Characterization of Prostate Adenocarcinoma
Source: Front Immunol. 2022 May 4;13:869759. doi: 10.3389/fimmu.2022.869759 (PMC9114358; doi:10.3389/fimmu.2022.869759)
Supplement: Supplementary file 8 [file Table_2.docx]

Supplemental table 2: The univariate Cox regression analysis on the prognostic values of m5C regulators for BCR in TCGA-PRAD cohort.

| **id** | **HR** | **HR.95L** | **HR.95H** | **pvalue** |
| --- | --- | --- | --- | --- |
| NSUN2 | 1.011787 | 0.993508 | 1.030402 | 0.207753 |
| NSUN3 | 1.012337 | 0.914139 | 1.121083 | 0.813797 |
| NSUN4 | 1.000027 | 0.92627 | 1.079658 | 0.999447 |
| NSUN5 | 1.014618 | 0.98802 | 1.041932 | 0.284302 |
| NSUN6 | 1.030536 | 0.968087 | 1.097013 | 0.345646 |
| NSUN7 | 0.992242 | 0.953502 | 1.032556 | 0.701507 |
| NOP2 | 1.019539 | 0.993092 | 1.04669 | 0.149013 |
| DNMT1 | 1.082061 | 1.037919 | 1.128081 | 0.000206 |
| DNMT3A | 1.068761 | 1.015034 | 1.125331 | 0.011503 |
| DNMT3B | 1.228196 | 1.123428 | 1.342734 | 6.23E-06 |
| TRDMT1 | 0.843921 | 0.634508 | 1.122449 | 0.243551 |
| ALYREF | 1.007986 | 0.999126 | 1.016924 | 0.077405 |
| YBX1 | 1.001682 | 0.999934 | 1.003433 | 0.059307 |
| TET1 | 0.869723 | 0.572862 | 1.320418 | 0.512326 |
| TET2 | 1.031032 | 0.941316 | 1.129298 | 0.510577 |
| TET3 | 1.02807 | 0.987568 | 1.070233 | 0.177034 |
| ALKBH1 | 0.951498 | 0.87231 | 1.037875 | 0.262102 |
