# Supplementary material for: Roles of m5C RNA Modification Patterns in Biochemical Recurrence and Tumor Microenvironment Characterization of Prostate Adenocarcinoma
Source: Front Immunol. 2022 May 4;13:869759. doi: 10.3389/fimmu.2022.869759 (PMC9114358; doi:10.3389/fimmu.2022.869759)
Supplement: Supplementary file 13 [file Table_7.docx]

Supplemental table 7: 33 genes were identified based on LASSO Cox regression algorithm.

| **Gene** | **Coef** |
| --- | --- |
| CLSTN3 | 0.10051 |
| TTYH3 | 0.314147 |
| SLC5A6 | 0.124479 |
| PLCG1 | 0.121703 |
| MGAT4B | 0.127688 |
| CPNE1 | 0.343099 |
| TXNL1 | -0.36546 |
| MDH1 | -0.15001 |
| PIK3R3 | 0.264311 |
| COMMD10 | -0.10354 |
| PRR14 | 0.727994 |
| MS4A7 | 0.295124 |
| OSBPL10 | 0.014852 |
| CCNG1 | -0.16758 |
| PRR7 | 0.001846 |
| MRPL39 | -0.10157 |
| VASH1 | 0.243953 |
| GTF2B | -0.90056 |
| TM4SF18 | 0.016262 |
| HSF4 | 0.068135 |
| TIAM1 | 0.021348 |
| PLXNB3 | 0.142981 |
| KBTBD6 | -0.12124 |
| TREM2 | 0.129774 |
| TPP1 | -0.76238 |
| RNASE1 | 0.254647 |
| SAP30 | 0.218891 |
| RGS5 | 0.01354 |
| GRIPAP1 | 0.051482 |
| HMOX1 | 0.014285 |
| FGD1 | 0.032518 |
| FBXO6 | 0.146869 |
| CA2 | 0.00917 |
